# Primary endodermal hemangiopericytoma/solitary fibrous tumor of the cervical spine: a case report and literature review

**DOI:** 10.1186/s12893-021-01399-6

**Published:** 2021-11-27

**Authors:** Liyun Wang, Jianbo Yu, Dongping Shu, Bin Huang, Yumin Wang, Luyuan Zhang

**Affiliations:** 1 Department of Neurosurgery, Shengzhou People’s Hospital (the First Affiliated Hospital of Zhejiang University Shengzhou Branch), Shengzhou, China; 2 Department of Neurosurgery, First Affiliated Hospital, School of Medicine, Zhejiang University, Hangzhou, China; 3 Department of Neurosurgery, Xinchang Hospital of Traditional Chinese Medicine, Shaoxing, China; 4 Department of Otolaryngology Head and Neck Surgery, Xiangya Hospital, Central South University, Changsha, China

**Keywords:** Hemangiopericytoma, Solitary fibrous tumor, Primary cervical spine tumor, Case report

## Abstract

**Background:**

Hemangiopericytoma (HPC), also known as solitary fibrous tumor (SFT), is a type of soft tissue sarcoma with a special aggressive behavior. The HPC/SFT is locally aggressive with possibility of late recurrence locally or distant extraneural metastasis. The most common location of this HPC/SFT is the lower extremities. The HPC/SFT in the central nervous system (CNS) is very rare, and compared with the brain, it is rarer in the spinal region. However, clinicians also lack an overall understanding of the diagnosis of HPC/SFT in the spinal cord.

**Case presentation:**

In this study, we report a rare case of primary cervical spine HPC/SFT in a 53-year-old woman. Two to three weeks before admission, she experienced pain and numbness in her left upper extremity. After computerized tomography (CT) and magnetic resonance imaging (MRI), a gross total resection was performed. Obvious neurological improvement was observed postoperatively. The pain and numbness in the patient's left upper limb were relieved subsequently. We then reviewed the literature on HPC/SFT, such as its clinical presentation, imaging characteristics, treatment, and follow-up.

**Conclusions:**

Diagnosis of HPC/SFT relies on magnetic resonance spectroscopy, enhanced CT, and MRI. Postoperative radiotherapy is strongly recommended to reduce the HPC/SFT recurrence. Immunohistochemical analysis can also help in the differential diagnosis. However; early and long-term follow-up is necessary for patients.

## Background

Hemangiopericytoma (HPC), also known as solitary fibrous tumor (SFT), is a type of soft tissue sarcoma with a special aggressive behavior that was first described by Klemperer and Rabin in 1931 [1]. The HPC/SFT can occur anywhere within existing capillaries. The most common location of HPC is the lower extremities, followed by the retroperitoneum and the head and neck regions [2]. Hemangiopericytoma in the central nervous system (CNS) is very rare, accounting for only 1–2% of all CNS tumors [3–7]. Compared with the brain, HPC/SFT occurs rarely in the spinal region [3]. Hemangiopericytoma/solitary fibrous tumor can be divided into WHO grades I to III pathologically, and the anaplastic HPC/SFT, which is WHO grade III [8]. The condition is locally aggressive with the possibility of late recurrence locally or distant extraneural metastasis [9]. The first case of spinal HPC/SFT was reported in 1958 [10]. To date, only approximately 100 cases of primary spinal HPC/SFT have been reported. In this study, we report a case of primary cervical spine HPC/SFT in a 53-year-old woman who presented with left upper limb pain and numbness. Magnetic resonance imaging (MRI) showed C6-7 levels of intradural extramedullary lesions, with a slightly hyperintense T2 weighted imaging (T2WI) signal like T1 weighted imaging (T1WI) in the surrounding small portion, low T1WI, and low T2WI signals without enhancement at the center. The spinal cord was compressed and pushed to the right. A three-dimensional reconstruction computerized tomography (CT) of the cervical spine revealed no obvious bone destruction. Herein, we report this case and reviewed the literature on the demographics, clinical presentation, imaging data, treatment, prognosis, and follow-up of HPC/SFT.

## Case presentation

A 53-year-old woman who was admitted with pain in the left upper extremity, which started two to three weeks before admission. The pain was paroxysmal and dull. The numerical rating scale (NRS) scores for pain ranged from 4 to 5. She underwent acupuncture and non-steroidal anti-inflammatory treatment, but her symptoms did not improve. Neurological examination revealed intact motor and sensory examinations throughout the upper and lower extremities, but revealed hyporeflexia on the left side of the body, which was pathologically positive. Cerebellar, Romberg, and gait examinations were normal. Disorders in the bowel and bladder were not observed.

**Fig. 1:**
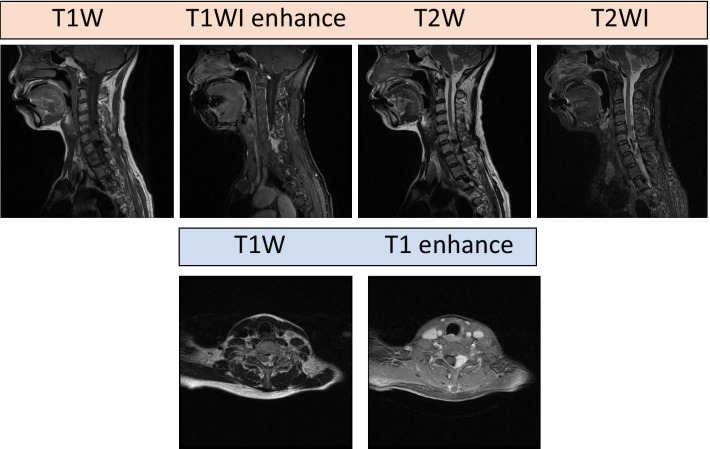
Magnetic resonance imaging of the patient. The left intervertebral foramen of C6-7, with an ill-defined margin that is 2.8 × 1.5 × 1.4 cm in size, and a slightly hyperintense T2WI signal like T1WI in the surrounding small portion, low T1WI and low T2WI signal without enhancement in the center. The spinal cord is compressed and pushed to the right. *T1* weighted imaging, T1WI; *T2* weighted imaging

The MRI after admission showed a spindle-shaped extradural lesion in the left intervertebral foramen of C6-7 with an ill-defined margin that is 2.8 × 1.5 × 1.4 cm in size, a slightly hyperintense T2WI signal like T1WI in the surrounding small portion, low T1WI, and low T2WI signals without enhancement at the center. The spinal cord was compressed and pushed to the right. A three-dimensional reconstruction CT of the cervical spine revealed no obvious bone destruction. (Fig. 1).

**Fig. 2:**
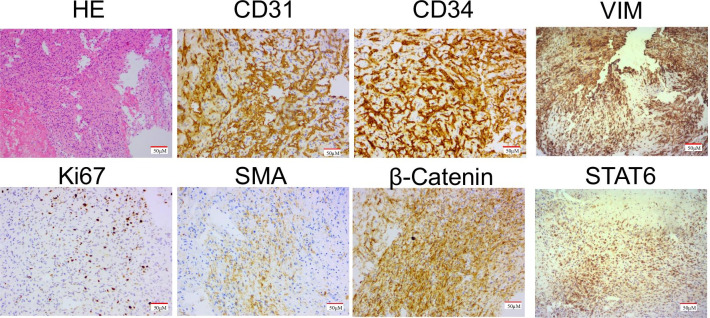
HE staining and IHC staining of tumor tissue. **A** Tumor cells were arranged in sheets and rich in blood vessels as shown in HE staining image. **B**–**F** Immunohistochemical study showing CD34 positive diffusely, β-catenin and CD31 vessels positive, SMA positive, Ki-67 15% positive. *HE* Hematoxylin and eosin, *IHC* immunohistochemical

According to the clinical and radiological examination results, it is preliminarily considered a neoplastic lesion with a high possibility of malignant behavior. Embolization was performed to reduce the intraoperative blood loss before surgery. Subsequently, spinal decompression was performed. When the dura mater was cut intraoperatively, a subdural tumor was observed on the left side of the cervical spinal cord, with a dark brown color and ill-defined boundary. The tumor in the spinal canal was completely resected in a piecemeal manner. A gross total resection was performed with no evidence of tumor in the tumor bed, suggesting the total tumor resection. Obvious neurological improvement was observed postoperatively. The pain and numbness in the patient's left upper limb were relieved. Pathological examination of the tumor tissue showed that the tumor cells had a spindle shape, mild cell morphology, rare nuclear division, diffuse hemangiopericymatoid distribution, and rich blood vessel supply (Fig. 2). Immunohistochemical analysis showed Ki-67 (about 15%), CD117 (−), S-100 (−), Desmin (−), CD34 ( +), SMA ( +), DOG-1(−), TTF-1 (−), EMA(-), PR(−), F8-R-Ag ( +), HMB45 (−), MelanA (−), TFE3 (−), CD31 ( +), HHV8 (−), Inhibina (−), β-Catenin ( +), GFAP (−), and CD68 ( +). Based on the above results, a diagnosis of HPC/SFT was made (Fig. 2).

Postoperative recovery was uneventful and without complications. The pain and numbness of the patient's left upper limb were relieved (NRS scores of 2–3). Her upper and lower limb motor function was not affected, and on the eight day after the surgery, the patient recovered and was able to ambulate with a neck collar. Due to the patient's subjective refusal of adjuvant radiotherapy and financial reasons, radiotherapy could not be administered after the surgery. The patient was advised to visit the hospital for a review in six months. However, because of COVID-19 and the patient's subjective reasons, she did not visit our hospital for the review. However, we followed up the patient by telephone severally, and the patient felt no significant discomfort or neurological symptoms related to HPC recurrence. Because the time was still short and the patient refused to receive an adjuvant radiotherapy, we will continue to follow her up on whether recurrence and neurological symptoms will occur subsequently.

## Discussion and conclusions

Most CNS HPC/SFTs occur in the cranial cavity. Spinal HPC/SFT is a rare and highly vascular mesenchymal neoplasm [1]. The HPC/SFT is characterized by aggressive and widespread migration [3]. In the past, because of the lack of typical clinical features and imaging manifestations, diagnosis and treatment have been a great challenge for clinicians. Herein, we report an uncommon case of primary cervical intradural extramedullary HPC/SFT and summarize our comprehensive literature review regarding this disease (Table 1).

**Table 1:** Summary of reported cases of primary spinal HPC

	Summary	Liu (9)	Das (11)	Yi (12)	Jia (13)	Wang (14)	Singla (15)
M/Average age	25/40.3	14/29.3	3/40.3	8/42.6	9/50.2	6/46.5	5/36.8
F/Average Age	20/33.9	12/36.8	2/24.5	3/41.3	11/39.2	10/36.7	5/32.6
Asia	2	26	/	11	20	16	/
Occident	42	/	5	/	/	/	10
Cervical segment	18	10	2	2	6	4	4
Thoracic segments	18	9	3	5	8	9	/
Lumbar segment	8	7	/	4	5	3	6
Sacral segment	1	0	/	/	1	/	/
Mean follow-up years(y)	4.03	6.57	2.15	3.39	3.19	8.03	5.43

**Fig. 3:**
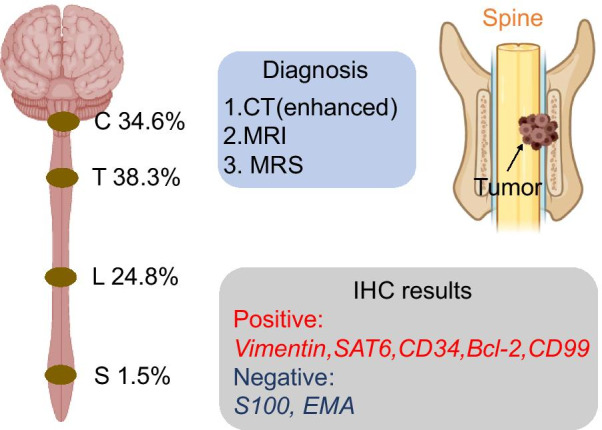
Summary of primary endodermal hemangiopericytoma/solitary fibrous tumor in the spinal cord

To the best of our knowledge, there have been 133 cases of primary spinal HPC/SFT reported up to the year 2020. These cases indicate that primary spinal HPC/SFT can occur at any age. The mean age of onset was 40.9 years old for males and 35.0 years old for females. There was no significant difference in morbidity between men and women (male/female ratio = 1.11:1). The incidence ratio of HPC/SFT in Asians and Occident is 1.32:1, suggesting that Asians are more susceptible. Furthermore, according to statistical data from our literature review, the thoracic spinal cord was the most common site of spinal lesions (38.3%), followed by the cervical and lumbar spinal cord (34.6 and 24.8%, respectively), and the lesions were rarely found in the sacral spinal cord (1.5%) (Fig. 3**)** [9, 15, 23].

The clinical symptoms of primary spinal HPC/SFT are nonspecific. They include pain, hypoesthesia, paraplegia, urinary dysfunction, extremity numbness and weakness, or a combination of the above, depending on the size and location of the tumor [9, 13, 16–22].

Diagnosis is usually difficult before surgery because HPC/SFT is a rare disease with nonspecific clinical features. The differential diagnoses included meningioma, malignant schwannoma, neuroblastoma, neurofibroma, metastases, solitary plasmacytoma, leukemia, sarcoidosis, lymphoma, and tuberculosis infection [16, 23–26]. The medical imaging of HPC/SFT has its characteristics, but has not been summarized in previous articles. In this study, we provide a summary. Enhanced CT and MRI are valuable imaging techniques for the diagnosis of HPC/SFT. The characteristic imaging features include a well-defined, oval, irregular or dumbbell-shaped solitary spinal mass, internal signal voids, marked and heterogeneous enhancement, with or without bone destruction on the CT and/or MRI [7, 9, 12, 18, 19, 27–33]. Besides, when the CT value of the tumor is greater than 161hu, suggesting a high vascular tumor, the diagnosis of HPC/SFT or angiomatous meningioma needs to be highly suspected [34]. Furthermore, magnetic resonance spectroscopy (MRS) can be used to distinguish HPC/SFT from meningiomas. Compared with meningiomas, HPC/SFT has a higher relative ratio of Myo, GLC, and GSH to Glu, while the relative ratios of CR, Gln, Ala, Gly, and Chocc to Glu in HPC/SFT are lower [35].

The five-year survival rate and local tumor control rate of HPC/SFT were 76%. Even after surgery and adjuvant radiotherapy, 29% of the tumors recurred [9, 17, 18, 33, 36]. There are three factors that can affect prognosis: total tumor resection, postoperative radiotherapy, and low tumor grade [29, 36–40]. To the extent possible, total resection is the preferred treatment for HPC/SFT. This seems to be a consensus. However, because HPC/SFT has marked vascularity, massive intraoperative bleeding is often an important factor affecting total tumor resection. Preoperative endovascular embolization of tumors has been proven to be effective in controlling intraoperative bleeding and may facilitate surgical resection of the lesions [10, 11, 23, 28, 40–47]. However, for patients at high risk for injury to the nerve root or dura mater, or those with type IB and III tumors [9, 11], it is usually difficult to perform total tumor resection without significant impact on the limb function or nerve injury. Therefore, total resection should not be overemphasized [9, 48–50]. Postoperative radiotherapy is necessary to prevent recurrence [4, 16, 18, 36, 40, 50–55]. The recurrence rates were 25.5% and 44.4% in patients with and without radiotherapy, respectively. Postoperative radiotherapy significantly reduced the recurrence rate (*P* < 0.05) (Table 2). The HPC/SFT is often aggressive, with a high recurrence rate and distant metastasis [18, 26, 41, 46]. Therefore, even if the primary lesion is well controlled, regular follow-up is necessary [9, 13, 18, 23, 48, 53].

**Table 2:** Summary of reported radiotherapy and non-radiotherapy of primary spinal HPC

	Recurrence	Summary	Liu(9)	Das(11)	Yi(12)	Jia(13)	Wang(14)	Singla(15)
With Radiotherapy	No	13	7	3	1	/	5	/
	Yes	3	17	1	/	/	1	/
	Unknown	4	/	/	/	14	/	6
Without Radiotherapy	No	15	/	1	8	/	3	/
	Yes	8	2	/	2	/	7	/
	Unknown	2	/	/	/	6	/	4

We reviewed 47 cases of HPC/SFT immunohistochemical analyses. To the best of our knowledge, this is the largest sample to date. We found that these lesions were typically positive for vimentin (n = 31/39, 79.5%), SAT6 (n = 23/26, 61.5%), CD34 (n = 35/47, 74.5%), Bcl-2 (n = 23/30, 76.7%), and CD99 (n = 17/19, 89.5%), and were negative for S100 (n = 5/27, 18.5%) and EMA(n = 0/27)[9–15]. This helps to distinguish between nerve sheath tumors and meningiomas [12, 55]. The Ki-67 index ranged from 1 to 80%. It was significantly higher in the WHO grade III group (13.1%) than in the WHO grade II group (7.0%) and group I (8.2%) (*P* < 0.05) (Table 3). We summarized that the clinical symptoms of HPC/SFT are atypical. The HPC/SFT imaging has certain characteristics in the MRS, enhanced CT, and MRI. Total resection is the best option, but preoperative embolization is necessary to reduce intraoperative bleeding when HPC/SFT is highly suspected.

**Table 3:**
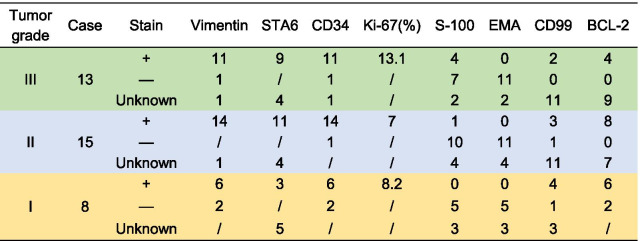
Summary of reported cases of Immunohistochemistry results of primary spinal HPC

Hemangiopericytoma, also known as solitary fibrous tumor, is a rare vascular-rich tumor with aggressive behavior and high recurrence. Because this was a very rare condition, it did not attract enough attention, and the preoperative preparation and evaluation were inadequate, which affected the total resection of the tumor. High vascularity of the tumor is associated with an increased risk of intraoperative bleeding, leading to significant morbidity and mortality. Therefore, we report a rare case of primary cervical HPC/SFT with a statistical analysis based on a review of the literature, emphasizing the importance of a correct diagnosis. Although the clinical symptoms are atypical, HPC/SFT imaging has certain characteristics on MRS, enhanced CT, and MRI. Summation would help in the diagnosis of HPC/SFT. To date, total resection is the best option, but preoperative embolization is necessary to reduce intraoperative bleeding when HPC/SFT is highly suspected. However, postoperative radiotherapy is strongly recommended to reduce HPC/SFT recurrence. Immunohistochemical analysis can also help in the differential diagnosis. Early and long-term follow-ups are also necessary.

## Data Availability

All relevant data are provided in the manuscript.

## Abbreviations

HPC: Hemangiopericytoma
SFT: Solitary fibrous tumor
CNS: Central nervous system
CT: Computerized tomography
MRI: Magnetic resonance imaging
MRS: Magnetic resonance spectroscopy
T1WI: T1 weighted imaging
T2WI: T2 weighted imaging

## References

[1] Klemperer P, Coleman BR (1992). Primary neoplasms of the pleura. A report of five cases. Am J Ind Med.

[2] Espat NJ, Lewis JJ, Leung D, Woodruff JM, Antonescu CR, Shia J, Brennan MF (2002). Conventional hemangiopericytoma: modern analysis of outcome. Cancer.

[3] Aaronson DM, Martinez Del Campo E, Boerger TF, Conway B, Cornell S, Tate M, Mueller WM, Chang EF, Krucoff MO (2021). Understanding variable motor responses to direct electrical stimulation of the human motor cortex during brain surgery. Front Surg..

[4] Kruse F (1961). Hemangiopericytoma of the meniges (angioblastic meningioma of Cushing and Eisenhardt). Clinico-pathologic aspects and follow-up studies in 8 cases. Neurology.

[5] Jääskeläinen J, Servo A, Haltia M, Wahlström T, Valtonen S (1985). Intracranial hemangiopericytoma: radiology, surgery, radiotherapy, and outcome in 21 patients. Surg Neurol.

[6] Kochanek S, Schröder R, Firsching R (1986). Hemangiopericytoma of meninges. I. Histopathological variability and differential diagnosis. Zentralbl Neurochir.

[7] Ackerman PD, Khaldi A, Shea JF (2011). Intradural hemangiopericytoma of the thoracic spine: a case report. Spine J.

[8] Louis DN, Ohgaki H, Wiestler OD, Cavenee WK, Burger PC, Jouvet A, Scheithauer BW, Kleihues P (2007). The 2007 WHO classification of tumours of the central nervous system. Acta Neuropathol.

[9] Liu H-G, Yang A-C, Chen N, Yang J, Qiu X-G, Zhang J-G (2013). Hemangiopericytomas in the spine: clinical features, classification, treatment, and long-term follow-up in 26 patients. Neurosurgery.

[10] Ciappetta P, Celli P, Palma L, Mariottini A (1985). Intraspinal hemangiopericytomas. Report of two cases and review of the literature. Spine.

[11] Dufour H, Métellus P, Fuentes S, Murracciole X, Régis J, Figarella-Branger D, Grisoli F (2001). Meningeal hemangiopericytoma: a retrospective study of 21 patients with special review of postoperative external radiotherapy. Neurosurgery.

[12] Pitlyk PJ, Dockery MB, Miller RH (1965). Hemangiopericytoma of the spinal cord: report of three cases. Neurology.

[13] Zhao Y, Zhao J-Z (2007). Clinical and pathological characteristics of primary intraspinal hemangiopericytoma and choice of treatment. Chin Med J.

[14] Shirzadi A, Drazin D, Gates M, Shirzadi N, Bannykh S, Fan X, Hunt L, Baron EM, King WA, Kim TT, Johnson JP (2013). Surgical management of primary spinal hemangiopericytomas: an institutional case series and review of the literature. Eur Spine J.

[15] Jia Q, Zhou Z, Zhang D, Yang J, Liu C, Wang T, Wu Z, Yang C, Wei H, Zhao J, Liu T, Zhou W, Yang X, Xiao J (2018). Surgical management of spinal solitary fibrous tumor/hemangiopericytoma: a case series of 20 patients. Eur Spine J.

[16] Kakimaru H, Matsusaki M, Sanada H, Iwata A, Uchio Y (2009). Dumbbell-type spinal solitary fibrous tumor with paraplegia. Orthopedics.

[17] Bohinski RJ, Mendel E, Aldape KD, Rhines LD (2004). Intramedullary and extramedullary solitary fibrous tumor of the cervical spine. Case report and review of the literature. J Neurosurg.

[18] Endo K, Komagata M, Ikegami H, Nishiyama M, Tanaka S, Imakiire A, Serizawa H (2003). Dumbbell-type solitary fibrous tumor in the cervical spine. J Orthop Sci.

[19] Mohammadianpanah M, Torabinejad S, Bagheri MH, Omidvari S, Mosalaei A, Ahmadloo N (2004). Primary epidural malignant hemangiopericytoma of thoracic spinal column causing cord compression: case report. Sao Paulo Med J.

[20] Hayashi Y, Uchiyama N, Hayashi Y, Nakada M, Iwato M, Kita D, Higashi R, Hirota Y, Kai Y, Kuratsu J-I, Hamada J-I (2009). A reevaluation of the primary diagnosis of hemangiopericytoma and the clinical importance of differential diagnosis from solitary fibrous tumor of the central nervous system. Clin Neurol Neurosurg.

[21] Rao S, Rajkumar A, Kuruvilla S (2008). Angiomatous meningioma: a diagnostic dilemma. Indian J Pathol Microbiol.

[22] Lee JK, Kim SH, Joo SP, Kim TS, Jung S, Kim JH, Lee JH (2006). Spinal metastasis from cranial meningeal hemangiopericytomas. Acta Neurochir.

[23] Yi X, Xiao D, He Y, Yin H, Gong G, Long X, Liao W, Li X, Sun L, Zhang Y, Zhang B (2017). Spinal solitary fibrous tumor/hemangiopericytoma: a clinicopathologic and radiologic analysis of eleven cases. World Neurosurg.

[24] Liu L, Yin B, Geng D-Y, Li Y, Zhang B-Y, Peng W-J (2014). Comparison of ADC values of intracranial hemangiopericytomas and angiomatous and anaplastic meningiomas. J Neuroradiol.

[25] Chou C-W, Hsu SPC, Lin S-C, Chen M-H, Shih Y-H, Lee L-S, Lin C-F (2009). Primary intradural hemangiopericytoma with intramedullary invasion. J Chin Med Assoc.

[26] Betchen S, Schwartz A, Black C, Post K (2002). Intradural hemangiopericytoma of the lumbar spine: case report. Neurosurgery.

[27] Fitzpatrick D, Mahajan J, Lewkowitz M, Black K, Setton A, Woldenberg R (2009). Intradural hemangiopericytoma of the lumbar spine: a rare entity. AJNR Am J Neuroradiol.

[28] Kashiwazaki D, Hida K, Yano S, Seki T, Iwasaki Y (2007). Subpial hemangiopericytoma with marked extramedullary growth: case report. Neurosurgery.

[29] Chen Q, Chen X-Z, Wang J-M, Li S-W, Jiang T, Dai J-P (2012). Intracranial meningeal hemangiopericytomas in children and adolescents: CT and MR imaging findings. AJNR Am J Neuroradiol.

[30] Sibtain NA, Butt S, Connor SEJ (2007). Imaging features of central nervous system haemangiopericytomas. Eur Radiol.

[31] Arai N, Mizutani K, Takahashi S, Morimoto Y, Akiyama T, Horiguchi T, Mami H, Yoshida K (2018). Preoperative assessment of pathologic subtypes of meningioma and solitary fibrous tumor/hemangiopericytoma using dynamic computed tomography: a clinical research study. World Neurosurg.

[32] Righi V, Tugnoli V, Mucci A, Bacci A, Bonora S, Schenetti L (2012). MRS study of meningeal hemangiopericytoma and edema: a comparison with meningothelial meningioma. Oncol Rep.

[33] Guthrie BL, Ebersold MJ, Scheithauer BW, Shaw EG (1989). Meningeal hemangiopericytoma: histopathological features, treatment, and long-term follow-up of 44 cases. Neurosurgery.

[34] Kim JH, Jung H-W, Kim Y-S, Kim CJ, Hwang S-K, Paek SH, Kim DG, Kwun BD (2003). Meningeal hemangiopericytomas: long-term outcome and biological behavior. Surg Neurol.

[35] Soyuer S, Chang EL, Selek U, McCutcheon IE, Maor MH (2004). Intracranial meningeal hemangiopericytoma: the role of radiotherapy: report of 29 cases and review of the literature. Cancer.

[36] Schiariti M, Goetz P, El-Maghraby H, Tailor J, Kitchen N (2011). Hemangiopericytoma: long-term outcome revisited. Clinical article J Neurosurg.

[37] Combs SE, Thilmann C, Debus J, Schulz-Ertner D (2005). Precision radiotherapy for hemangiopericytomas of the central nervous system. Cancer.

[38] Brass SD, Guiot M-C, Albrecht S, Glikstein R, Mohr G (2004). Metastatic hemangiopericytoma presenting as an epidural spinal cord lesion. Can J Neurol Sci.

[39] Chang C-C, Chang Y-Y, Lui C-C, Huang C-C, Liu J-S (2004). Meningeal hemangiopericytoma with delayed multiple distant metastases. J Chin Med Assoc.

[40] Cizmeli MO, Ilgit ET, Ulug H, Erdogan A (1992). A giant paraspinal hemangiopericytoma and its preoperative embolization. Neuroradiology.

[41] Cole CD, Schmidt MH (2009). Hemangiopericytomas of the spine: case report and review of the literature. Rare Tumors.

[42] Muraszko KM, Antunes JL, Hilal SK, Michelsen WJ (1982). Hemangiopericytomas of the spine. Neurosurgery.

[43] Musacchio M, Mont’Alverne F, Belzile F, Lenz V, Riquelme C, Tournade A (2003). Posterior cervical haemangiopericytoma with intracranial and skull base extension. Diagnostic and therapeutic challenge of a rare hypervascular neoplasm. J Neuroradiol.

[44] Salvati M, Ciappetta P, Artico M, Raco A, Fortuna A (1991). Intraspinal hemangiopericytoma: case report and review of the literature. Neurosurg Rev.

[45] Das A, Singh PK, Suri V, Sable MN, Sharma BS (2015). Spinal hemangiopericytoma: an institutional experience and review of literature. Eur Spine J.

[46] Li Z, Deng Y, Li Z, Wang T, Gao J, Zhou W, Li Y, Wang Y (2019). Primary epidural hemangiopericytoma of the thoracic spine: case report and literature review. J Clin Neurosci.

[47] Ijiri K, Yuasa S, Yone K, Matsunaga S, Ryoki Y, Taniguchi N, Yonezawa S, Komiya S (2002). Primary epidural hemangiopericytoma in the lumbar spine: a case report. Spine.

[48] Peng Z, Wang Y, Wang Y, Fan R, Gao K, Zhang H, Jiang W (2021). Comparing the effectiveness of endoscopic surgeries with intensity-modulated radiotherapy for recurrent rT3 and rT4 nasopharyngeal carcinoma: a meta-analysis. Front Oncol..

[49] Uemura S, Kuratsu J, Hamada J, Yoshioka S, Kochi M, Ushio Y, Nakahara T, Kishida K (1992). Effect of radiation therapy against intracranial hemangiopericytoma. Neurol Med Chir.

[50] Galanis E, Buckner JC, Scheithauer BW, Kimmel DW, Schomberg PJ, Piepgras DG (1998). Management of recurrent meningeal hemangiopericytoma. Cancer.

[51] Kaur J, Pandit S, Sharma MC, Julka PK, Rath GK (2015). Intradural extra medullary hemangiopericytoma of dorsal spine. Childs Nerv Syst.

[52] Ecker RD, Marsh WR, Pollock BE, Kurtkaya-Yapicier O, McClelland R, Scheithauer BW, Buckner JC (2003). Hemangiopericytoma in the central nervous system: treatment, pathological features, and long-term follow up in 38 patients. J Neurosurg.

[53] Mekni A, Kourda J, Chelly I, Ferchichi L, Bellil K, Hammouda KB, Kchir N, Zitouna M, Khaldi M, Haouet S (2008). Hemangiopericytoma in the central nervous system. A study of eight cases. Neurochirurgie.

[54] Wu W, Shi J-X, Cheng H-L, Wang H-D, Hang C-H, Shi Q-L, Yin H-X (2009). Hemangiopericytomas in the central nervous system. J Clin Neurosci.

[55] Zhang P, Hu J, Zhou D (2014). Hemangiopericytoma of the cervicothoracic spine: a case report and literature review. Turk Neurosurg.

